# Early detected femoral neck insufficiency fracture in a patient treated with long-term bisphosphonate therapy for osteoporosis: A need for MRI

**DOI:** 10.1016/j.ijscr.2020.04.003

**Published:** 2020-05-07

**Authors:** Yoon Jae Seong, Jong Ki Shin, Won Ro Park

**Affiliations:** Department of Orthopaedic Surgery, Dongeui Medical Center, 62 Yangjung-ro, Jin-gu, Busan, 47227, South Korea

**Keywords:** Insufficiency fracture, Femoral neck, Bisphosphonate, Magnetic resonance imaging

## Abstract

**Introduction:**

Atypical femoral fracture is an uncommon complication of prolonged use of bisphosphonates, mainly occurring in the femoral shaft. We report a case of an atraumatic insufficiency fracture of femoral neck after four years of treatment with a bisphosphonate.

**Presentation of case:**

A 71-year-old female presented with history of pain on Lt. hip and difficulty in walking. Although there was no fracture on the radiographs, diagnosis could be made early using magnetic resonance imaging (MRI) scans. She was treated by internal fixation using cannulated screws.

**Discussion:**

Because bisphosphonate prevents bone healing and remodeling, it is difficult to accomplish the bony union despite of proper treatment in patients who have fractured after long-term use of bisphosphonate.

**Conclusion:**

A high suspicion index and early diagnosis through the use of MRI are essential for the successful treatment of these fractures.

## Highlights

• If long term bisphosphonate users complain of non-traumatic hip pain, medical examination should be taken with care.
• Even though no fractures are identified in their radiographs, it is recommended to check an MRI.
• If the fracture can be identified before cortical breakage, the treatment success rate for the fracture will be high.

## 1 Introduction

Osteoporosis is a disorder characterized by low bone density and impaired bone strength which is an important risk factor for fracture in older adults [1]. Bisphosphonate is one of the medicines for osteoporosis and has been used for many years to reduce bone loss and prevent fractures. Many studies have been reported that bisphosphonate elevates the bone mineral density of osteoporosis patients and is effective in preventing fractures [2,3]. However, as a result of long-term follow-up of patients using bisphosphonate, two major complications, osteonecrosis of jaw and atypical femoral fracture, were identified [4]. In addition, femoral neck insufficiency fracture has been reported in several studies, those studies indicated difficulties in treating femoral neck insufficiency fracture after prolonged use of bisphosphonate [[5], [6], [7], [8]]. We accordingly report a case of early diagnosis and successful treatment of patients with femoral neck insufficiency fracture after prolonged use of bisphosphonate. The work has been reported in line with the SCARE criteria [9].

## 2 Presentation of case

**Fig. 1:**
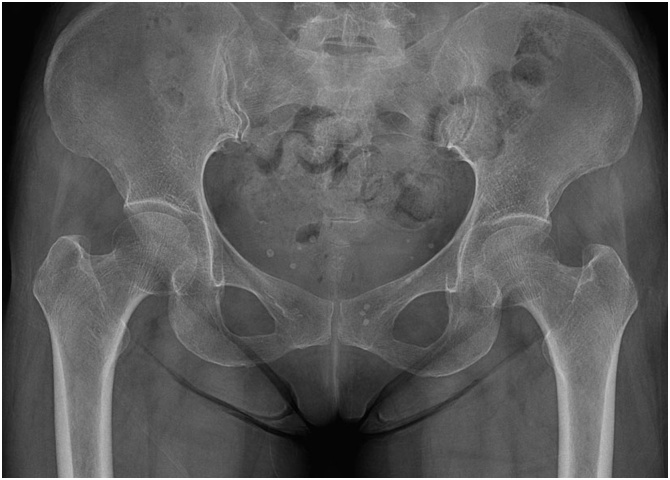
Radiographs of patient’s pelvis showed no evidence of fractures on Lt. hip.

**Fig. 2:**
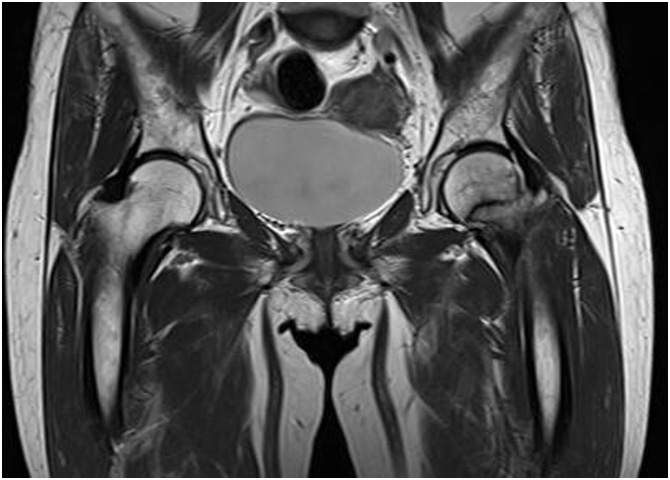
The MRI scan showed the incomplete linear fracture of the Lt. femoral neck.

**Fig. 3:**
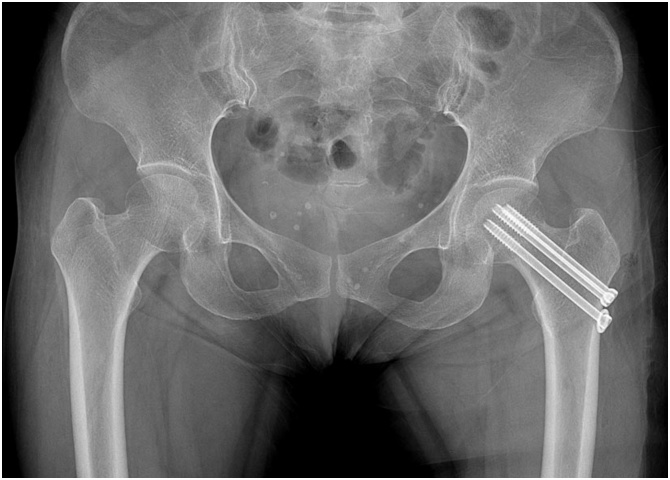
The patient underwent internal fixation using 3 cannulated screws.

A 71-year-old female presented to our hospital with history of pain on Lt. hip and difficulty in walking for the past few days. There was no definite history of significant trauma like falls. Clinical examination revealed no deformities of both legs and severe pain on passive movement of Lt. hip. And a positive Patrick’s test result on the Lt. hip. Radiographs of her pelvis showed no evidence of fractures on Lt. hip (Fig. 1). According to her medical history, she had been diagnosed as having osteoporosis (T-score −3.0 at spine and −2.0 at femoral neck), and had received treatment with the risedronate (Actonel EC) 35 mg once a week through oral administration for 4 years. She was admitted in to ward for pain control. A magnetic resonance imaging (MRI) scan of the pelvis including both hips was carried out to find the cause of on-going pain and also to rule out insufficiency femoral fractures. The MRI scan showed the incomplete linear fracture of the femoral neck (Fig. 2). The patient underwent internal fixation using 3 cannulated screws, and the postoperative radiograph was satisfactory (Fig. 3). The patient was recommended to walk using crutches for 1month, and the postoperative period was uneventful. Six weeks after surgery, she could ambulate independently without pain. At 3 months follow up, she could still ambulate well without Lt. hip pain.

## 3 Discussion

We report a case of femoral neck insufficiency fracture without trauma in a patient treated with long term bisphosphonate. Insufficiency fractures are a type of stress fracture, which are the result of normal stresses on abnormal bone. Insufficiency fracture is defined as an injury that occurs when minimal stress is applied to abnormal bone characterized by decreased elastic resistance [10]. They occur in elderly patients or in postmenopausal women with osteoporotic bone. Insufficiency fractures occur most frequently in the weight bearing bones like sacrum, tibia [10]. As with our report, there have been several studies that report insufficiency fractures in the femoral neck after long term treatment of bisphosphonate [[5], [6], [7], [8]].

Antiresorptive drugs such as bisphosphonate increase bone mass by inhibiting osteoclast activity and reducing bone resorption. This mechanism increases the bone mineral density (BMD) and reduces the chance of fracture. Although several factors such as bone structure, bone remodeling, bone quality are related to fractures, BMD is a strong predictor of fracture [11].

Bisphosphonates interfere with osteoclast activity and thus decrease the rate of bone resorption.

At the same time, bisphosphonate also plays a role in inhibiting osteoblast activity. As a result, bone resorption and bone formation decrease simultaneously. With regard to fractures, a decrease in bone resorption increases bone mass and prevents fractures. On the other hand, new bone formation is reduced, the bone quality worsens. This changed bone like osteopetrosis is easily broken to stress. Whyte et al. described bisphosphonate-induced osteopetrosis (marble bone disease) in a 12-year-old boy [12]. Prolonged treatment with high doses of pamidronate resulted in findings characteristic of osteopetrosis, including increased bone density and defective remodeling. Increased BMD following bisphosphonate use reduces the overall incidence of fractures in patients with osteoporosis. However, changed bony conditions increase the incidence of nonspecific fractures like atypical femoral shaft fracturs and femoral neck insufficiency fractures [4].

There is a difference in the rate of uptake between trabecular and cortical bones [13]. Differential uptake by trabecular and cortical sites depends on delivery via blood and may depend on the relative bone affinity of each bisphosphonates [14]. Previous work on skeletal distribution has predominantly focused on trabecular bone as this is where bisphosphonates produce their most prominent skeletal effect [13]. Although bisphosphonates also significantly affect cortical bone by reducing porosity through suppression of intracortical remodeling, the effects and side effects of using bisphosphonate will be more prominent in the trabecular bone than the cortical bone.

Femoral neck area can be stressed due to the morphological characteristics of the bone structure in stance loading conditions [15]. When stance loading is applied to the femoral neck, bending force is used repeatedly. Thus, the femoral neck area is more easily broken by minor trauma in osteoporotic state. In patients treated with long term bisphosphonate, bone conditions altered by the mechanism described above cause atypical fractures in the femoral neck area. In cases that have been reported femoral neck insufficient fractures to long term bisphosphonate users, most of the fracture site was superior cortex of the femoral neck [[5], [6], [7], [8]]. Human femurs have thinner superior than inferior cortices at the mid-femoral neck region. In addition, elderly femurs have marked thinning in the superior regions but have thicker cortices compared with young femurs [16]. Although this case did not progress to cortical breakage, we could confirm the cancellous bone fracture by MRI.

Several cases of insufficiency femoral fracture have been reported in patients with osteoporosis medications other than bisphosphonate. Among them, denosumab is an antiresorptive drug that can replace bisphosphonate and has been used in several years. Paparodis et al. reported an unusual, nontraumatic subtrochanteric insufficiency fracture in a patient treated with the antiresorptive agent denosumab [17].

Fractures are usually diagnosed only by radiographs. However, it is impossible to diagnose a fracture by radiographs if the cortical bone breakage has not progressed like in femoral neck insufficiency fracture. MRI can diagnose femoral neck insufficiency fracture that has not progressed to cortical bone breakage and can be identified by low signal intensity lines on T1 images. Considering the possibility of insufficiency femoral neck fracture, an early MRI test is necessary for the long-term bisphosphonate patients complaining of hip pain.

Bisphosphonate has produced sustained reduction of bone remodeling and osteogenesis, and this may have negative effect on bony union of fracture site [2]. Odvina et al. found a lack of bone formation and a delay of bone union in an iliac crest examination of nine cases for which patients were prescribed long-term treatment with alendronate and experienced abnormal stress fractures. Many studies have described the difficulty of treating bisphosphonate induced femoral neck insufficiency fracture [[5], [6], [7], [8]]. Femoral neck insufficiency fracture which progressed to cortical bone breakage, is difficult to obtain an union even after proper internal fixation surgery in long-term bisphosphonate users. Persistent pain due to nonunion of the fracture site will require artificial joint replacement surgery in the future.

Through this case and previous reports, the authors propose the following methods to improve the success rate of femoral neck insufficiency fracture treatment in the long-term bisphosphonate users. If long term bisphosphonate users complain of non-traumatic hip pain, medical examination should be taken with care. Even though no fractures are identified in their radiographs, it is recommended to check an MRI. If the fracture can be identified before cortical breakage, the treatment success rate for the fracture will be high. It should be noted that the success rate of internal fixation using screws generally decreases in the case of femoral neck insufficiency fracture that have been confirmed to cortical breakage. In some cases of cortical breakage, joint replacement surgery may need to be considered as a primary treatment option. In case of screw fixation surgery, use of osteogenic agents such as teriparatide and romosozumab may be considered.

## 4 Conclusion

The diagnosis of femoral neck insufficiency fracture can be missed easily by simple radiographs alone. If patients treated by long-term bisphosphonate have hip pain that persists without a history of significant trauma or unusual increase in daily activity, femoral neck insufficiency fractures should be considered. And an immediate MRI assessment will be needed for an early diagnosis and better clinical outcome.

## Declaration of Competing of Interest

The authors declare no conflict of interest.

## Sources of funding

We have nothing to declare.

## Ethical approval

This is exempt from ethnical approval in our institution.

## Consent

Informed consent was obtained from the patient for publication of this case report and accompanying figures.

## Authors contribution

Yoon Jae Seong: Conception and design the study, analysis and interpretation of data.

Jong Ki Shin: Acquisition of data, drafting the article.

Won Ro Park: Performing the surgery, Conception and design the study, Final approval of the version to be submitted.

## Registration of research studies

Not applicable.

## Guarantor

Won Ro Park.

## Provenance and peer review

Editorially reviewed, not externally peer-reviewed.
